# Conditioned Medium from Early-Outgrowth Bone Marrow Cells Is Retinal Protective in Experimental Model of Diabetes

**DOI:** 10.1371/journal.pone.0147978

**Published:** 2016-02-02

**Authors:** Diego A. Duarte, Alexandros Papadimitriou, Richard E. Gilbert, Kerri Thai, Yanling Zhang, Mariana A. B. Rosales, José B. Lopes de Faria, Jacqueline M. Lopes de Faria

**Affiliations:** 1 Renal Pathophysiology Laboratory, Investigation on Diabetes Complications, Faculty of Medical Sciences, State University of Campinas (UNICAMP), Campinas, Brazil; 2 Keenan Research Centre of the Li KaShing Knowledge Institute, St. Michael’s Hospital, University of Toronto, Toronto, Ontario, Canada; Indiana University College of Medicine, UNITED STATES

## Abstract

Bone marrow-derived cells were demonstrated to improve organ function, but the lack of cell retention within injured organs suggests that the protective effects are due to factors released by the cells. Herein, we tested cell therapy using early outgrowth cells (EOCs) or their conditioned media (CM) to protect the retina of diabetic animal models (type 1 and type 2) and assessed the mechanisms by *in vitro* study. Control and diabetic (db/db) mice (8 weeks of age) were randomized to receive a unique intravenous injection of 5×10^5^EOCs or 0.25 ml thrice weekly tail-vein injections of 10x concentrated CM and Wystar Kyoto rats rendered diabetic were randomized to receive 0.50 ml thrice weekly tail-vein injections of 10x concentrated CM. Four weeks later, the animals were euthanized and the eyes were enucleated. Rat retinal Müller cells (rMCs) were exposed for 24 h to high glucose (HG), combined or not with EOC-conditioned medium (EOC-CM) from db/m EOC cultures. Diabetic animals showed increase in diabetic retinopathy (DR) and oxidative damage markers; the treatment with EOCs or CM infusions significantly reduced this damage and re-established the retinal function. In rMCs exposed to diabetic milieu conditions (HG), the presence of EOC-CM reduced reactive oxygen species production by modulating the NADPH-oxidase 4 system, thus upregulating SIRT1 activity and deacetylating Lys-310-p65-NFκB, decreasing GFAP and VEGF expressions. The antioxidant capacity of EOC-CM led to the prevention of carbonylation and nitrosylation posttranslational modifications on the SIRT1 molecule, preserving its activity. The pivotal role of SIRT1 on the mode of action of EOCs or their CM was also demonstrated on diabetic retina. These findings suggest that EOCs are effective as a form of systemic delivery for preventing the early molecular markers of DR and its conditioned medium is equally protective revealing a novel possibility for cell-free therapy for the treatment of DR.

## Introduction

With the incidence of diabetes increasing at an alarming rate, the number of people with diabetic retinopathy (DR) is expected to grow from 126.6 million to 191 million by 2030 [1]. The known pathways, including oxidative stress, increased formation of AGEs, and activation of protein kinase-C and hexosamine pathways [2,3], appear to cross-talk. For this reason, new strategies to prevent these devastating complications are needed.

Cellular therapies for the treatment of degenerative retinal diseases such as DR and age-relatedmacular degeneration have been developed recently using a variety of cell types, including pluripotent stem cell- retinal pigment epithelium (RPE), bone marrow- or umbilical cord-derived mesenchymal stem cells, foetal neural or retinal progenitor cells, and stem cell-derived adult RPE [4–6]. Among many proposed cell types, circulating or bone marrow-derived endothelial progenitor cells (EPCs) have been the main cells used in clinical trials [7,8]. The mechanisms by which these cells exert their beneficial effects remain unclear, and studies have documented only minimal retention of administered cells within organs that have nevertheless sustained functional and structural improvements [9,10]. So-called early out growth cells (EOCs), defined by theidentification of their cell surface markers, have gained attention for their secretory feature of releasing antifibrotic and anti-oxidative stress factors [11–15]. Gilbert and colleagues described a successful treatment for diabetic nephropathy using a single infusion of EOCs in an experimental model of type 2 diabetes mellitusmice [14]. Similar findings were demonstrated in db/db mice treated with conditioned medium from EOCs, suggesting that the effects were mediated through released factors [15]. Mass spectrometric analysis of the EOC-conditioned medium (EOC-CM) identified proteins that regulate cellular functions implicated in fibrosis and proteins involved in stress responses, such as HSP-19, glutathione S transferase (GST1), peroxiredoxin, superoxide dismutase (SOD), thioredoxin, and heme oxygenase-1 [15]. These results indicate that EOCs display a great capacity for secreting soluble factor(s) with potent antioxidant activity that, when injected intravenously, replicate the salutary effects of the cells themselves.

Muller cell, the predominant glial cell in the retina, has its cell body residing in the inner nuclear layer and span all retinal layers, interacts with neighbouring neurons, being part of the outer and inner limiting membranes [16]. Due to this, Muller cells monitor the retinal structure and function. Muller cell processes wrap around retinal blood vessels thus controlling retinal barrier [17] but also mantain neurons by releasing trophic factors and recycling neurotransmitters and controlling ionic balance in the extracellular space [18]. These two both features are present in the pathogenesis of DR. Under sustained stress, as observed in diabetes, the Muller cells produce pro-inflamatory cytokines to restore the retinal homeostasis [19] upregulating glial fibrillary acidic protein (GFAP) [18] and VEGF [20] leading to a glial reaction and blood barrier hyper-permeability [21].

Sirtuins (SIRTs) are a family of deacetylases that require NAD+ as a cofactor for the deacetylation reaction. SIRT1 has been shown to play a role in gene silencing, apoptosis, senescence, and aging [22,23]. As it is NAD+ dependent, SIRT1 acts in some loci by removing the acetyl group from the histonechromatin [23], but it can also suppress other proteins by deacetylation, including the nuclear factor (NF)κB-p65subunit protein [24]. Under certain stimuli, such as reactive oxygen species (ROS), SIRT1 is inactivated, thus suppressing its deacetylase activity [25]. This redox-sensitive particularity of SIRT1 is explained by a reduction in substrate NAD+ [26] or by the post-translational modification of the SIRT1 protein [27]. Our group recently described the mechanism of glial reaction in Müller cells under diabetic conditions through acetylation of the NFκB-p65subunit due to impairment of SIRT1 activity [28].

In light of this evidence, the aim of this study was to investigate whether cellular therapy using intravenously injected cultured EOCs or their conditioned medium (CM) protect the retina of db/db mice and of experimentally diabetic WKY rats from the effects of the diabetic environment. To address the possible mechanism involved in the observed *in vivo* findings, retinal Müller cells (rMC) were exposed to high glucose (HG) and treated with CM from db/m EOCs (EOC-CM). Our results indicate that the intravenous infusion of EOCs or their CM mitigates the early DR markers, namely GFAP and VEGF, and oxidative markers in diabetic retinas of db/db mice and fully restored retinal function (evaluated through eletrophysiological means) in diabetic WKY rats. In rMCs exposed to HG, EOC-CM led to a decrease in ROS production via NOX4 system. The equitable redox status re-establishes SIRT1 activity through prevention of the posttranslational modification of the SIRT1 molecule, which in turn deacetylates Lys-310-p65-NFκB and decreases glial reaction, preventing the early DR markers.

## Methods

### Animal model and experimental design

#### Protocol 1

The animal study complied with the ARVO Statement for the Use of Animals in Ophthalmic and Vision Research. The Jackson Laboratory (Bar Harbor, ME) provided 36 male, diabetic db/db (BKS.Cg-Dock7 m +/+ Leprdb/J) 6-week-old and 12 age-matched db/m (Dock7 m+/+ Leprdb; heterozygote from the same colony) mice. At 8 weeks of age, the db/db mice were randomized to receive a tail-vein injection of medium only (DPBS saline; n = 4) or 5x10^5^ EOCs derived from db/m mice (n = 5) (study design A) or to receive 0.25 ml thrice weekly tail-vein injections of either: (1) EBM-2 medium (EBM-2, n = 8), or (2) 10X concentrated CM generated from EOCs derived from db/m mice (db/m CM, n = 10) (study design B). The db/m mice served as non-diabetic controls (n = 5). The mice were housed in a temperature-controlled room (22°C) with a 12h:12h light:dark cycle with free access to food and water. All animal studies were approved by the St. Michael’s Hospital Animal Ethics Committee in accordance with the Guide for the Care and Use of Laboratory Animals (NIH publ. no. 85–23, revised 1996). Four weeks after both treatments, the animals were euthanized, samples were collected, and retinal tissues were harvested.

#### Protocol 2

This study protocol was approved by the local committee for ethics in animal research (CEEA/IB/UNICAMP, protocol number 3551–1). Experimental diabetes was induced in 6-week-old male WKY by intravenous injection of streptozotocin (60 mg/kg in 0.5-M sodium citrate buffer, pH4.5; Sigma, St. Louis, MO, USA); the control animals received vehicle alone. Blood glucose levels were measured 72 h after the injection and values ≥15 mmol/L were considered diabetic. The animals were randomized to receive a tail-vein injection of 0.5 ml thrice weekly tail-vein injections for 2 weeks of either: (1) EBM-2 (WKY-DM+EBM-2, n = 4), or (2) 10X concentrated CM generated from EOCs derived from WKY (WKY-DM+CM, n = 4). The WKY rats diabetic untreated (WKY-DM, n = 4) and non-diabetic (WKY-CT, n = 5) served as experimental controls. The rats were housed in a temperature-controlled room (22°C) with a 12h:12h light:dark cycle with free access to food and water. Two weeks of the treatment, the animals were submitted to full flash electroretinography test (ERG).

#### Metabolic functional parameters

Body weight and blood glucose (Accu-check Advantage, Roche, Mississauga, ON) were determined bi-weekly.

#### Full-flash ERG recording

Retinal function was measured at the end of the study using the UTAS-E3000 system (LKC Technologies Inc., Gaithersburg, MD, USA) as previously described [28]. The pupils were dilated with tropicamide (Mydriacyl 0.5%; Allergan, Irvine, CA, USA). General anesthesia was induced with ketamine and xylazine (75 and 7.5 mg/kg, respectively) under dim red illumination (*λ*max = 650 nm). The measurements were taken after overnight dark adaptation. An intensity-response series was recorded using a series of ganzfeld flashes with 0.25 cd.s/m^2^ intensity. Recordings were amplified and digitized using a 24-bit A/D converter band passed from 0.3 to 300 Hz with a 50-Hz notch filter.

#### Tissue preparation and histology

Mice were euthanized 4 weeks after the initiation of the treatments. Eyes were collected and fixed in 10% neutral buffered formalin, followed by embedding in cryostat matrix (Tissue-Tek, Sakura, Kobe, Japan) or snap freezing in liquid nitrogen. Formalin-fixed eyes were routinely processed, embedded in paraffin, and sectioned.

#### Bone marrow harvesting and cell culture

The EOCs were cultured as described [12,29]. To produce the EOCs, bone marrow cells were collected from the femora and tibiae of 3- to 4-week-old male db/m mice or WKY rats and cultured in EGM-2 (Lonza, Walkersville, MD) at 37°C with 5% CO_2_ for 7–10 days when the total cell number reached ~2 x 106 per culture dish. An EOC-conditioned medium was then generated by incubating the EOCs with serum-free endothelial basal medium-2 (EBM-2) (Lonza, Walkersville, MD) for 24 hours. The medium was collected, centrifuged, and concentrated 10-fold using a centrifuge filtration column (Millipore, Billerica, MA). EOC CM was then filtered using a 0.45 μm filter (Millipore, Billerica, MA). EBM-2 medium (Lonza, Walkersville, MD) was concentrated and filtered as a control. Aliquots of the concentrated CM and EBM-2 were stored at -80°C.

#### EOC-CM generation

EOC-CM was generated as previously described [12,14]. The medium from subconfluent EOCs cultivated in a serum-free EBM-2 (Lonza) for 24 h was collected and used for *in vitro* experiments. Serum-free EBM-2 medium served as a control.

#### Immunohistochemistry in retinal tissues

Retinal sections (4 μm) were incubated with antibody against GFAP (DakoCytomation, Carpinteria, CA), VEGF (Santa Cruz Biotechnology, Santa Cruz, CA), nitrotyrosine (NT) (Upstate Biotechnology, Lake Placid, NY), 8-hydroxy-deoxiguanosine (8-OHdG) (JaICA;*NikkenSEIL Co*. Ltd, Shizuoka, Japan), and SIRT1 (Cell Signaling Technology, Danvers, MA) overnight at 4°C. The slides were then incubated with the appropriate secondary antibodies. The semi quantitative analyses were performed using the Leica Application Suite (Leica Microsystems, Wetzlar, Germany) in nine non-consecutive retinal sections divided among three slides per animal, per group, under high-powered microscopic viewing (×400) (Zeiss, Jena, Germany).

### *In vitro* studies

#### Transformed rat retinal Müller cell line (rMC-1)

Vijay J. Sarthy, PhD (Northwestern University, Evanston, IL) kindly provided the rMC-1, which was grown in DMEM-containing 5 mM of glucose, 10% foetal bovine serum (FBS), 24 mmol/L of NaHCO_3_, 10 mmol/L of HEPES, and 10.000 U/L of penicillin/streptomycin. The cells were maintained in a humidified incubator at 37°C and 5% CO_2_. At 70% of confluence, rMC-1 cell cultures were serum-starved to 1% FBS, then exposed for 24h with DMEM with 5.5 mmol/L glucose (NG), DMEM with 25 mmol/L glucose (HG), DMEM with 25 mmol/L glucose plus 10% of 10X concentrated db/m EOC-CM (HG+CM), or with other treatments as specified; DMEM with 5.5 mmol/L glucose plus 19.5 mmol/L of mannitol (MAN) was used as an osmotic control. The cytotoxicity of the treatments on rMCs was determined by thiazolyl blue tetrazolium bromide (MTT) colorimetric assay [30].

#### Transient transfection with siRNAs

The small interfering RNA (siRNA) duplexes and scrambled siRNA corresponded to rat SIRT1 (Santa Cruz Biotech) and rat NOX4 (Dharmacon, Lafayette, CO). The transient transfection of siRNAs was carried out using Lipofectamine® transfection reagent (Life technologies Carlsbad, CA [31].

#### Immunofluorescence in Müller cells

The immunofluorescence was performed as described [28]. The cells were incubated with anti-GFAP, anti-VEGF, or anti-SIRT1 antibodies (Santa Cruz) followed by the appropriate secondary antibody (Santa Cruz). Afterward, the sections were rinsed and cover-slipped with VECTASHIELD®, which is used for nuclei staining (Vector Laboratories, Burlingame, CA). The slides were examined under a scanning microscope (CLSM; Zeiss) with appropriate emission filters for fluorescein isothiocyanate 1 and rhodamine. Digital images were captured using AxioVision software (Zeiss). Negative controls were established by omitting the primary antibody.

#### Western blot

Retinal protein extraction or total rMC-1 lysate were incubated with anti-GFAP (Santa Cruz Biotech), anti-NT (Upstate Biotech), anti-SIRT1 (Santa Cruz), anti-acetylated-lys310-RelA/p65-NF-κB (Assay Biotechnology, Sunnyvale, CA), anti-p65 subunit NF-κBcomplex (Santa Cruz), and anti-NOX4 (Santa Cruz). Immunoreactive bands were visualized using the enhanced chemiluminescence method (Super Signal CL-HRP Substrate System; Pierce, Rockford, IL). Exposed films were scanned with a densitometer (Bio-Rad, Hercules, CA) and analysed quantitatively with Multi-Analyst Macintosh^®^ Software for Image Analysis Systems Bio-Rad. As an internal control for protein loadings, the membranes were hybridized against beta-actin (Santa Cruz). At least three independent experiments were carried out.

#### SIRT1 activity

SIRT1 activity was measured with a Fluorescent Activity Assay/Drug Discovery Kit designed to measure lysyl-deacetylase (Enzo Life Sciences, Farmingdale, NY). NAD^+^-dependent deacetylation of the substrate by recombinant human SIRT1 sensitized it to Developer II, which then generated a fluorophore. The fluorophore was excited with 360 nm light, and the emitted light was detected on a fluorometric plate reader (Synergy™ Mx;BioTek, Winooski, VT).

#### Intracellular ROS levels measured by H2DCF-DA

Intracellular ROS levels were measured by H_2_DCF-DA. Relative fluorescence was measured using a fluorescence plate reader (Synergy™ Mx) at excitation and emission wavelengths of 485 and 528 nm, respectively. The relative fluorescence values were corrected by the number of cells in each treatment.

#### Covalent modification of SIRT1

Immunoprecipitation was performed as before with minor modifications [32]. 500 μg of total rMC lysate diluted in extraction buffer were immunoprecipitated with rabbit anti-SIRT1 (Santa Cruz) using Protein A/G agarose beads. The samples were subjected to Western blotting against nitrotyrosine (Upstate).

To determine the carbonylation of SIRT1, blots were probed first with anti-SIRT1 antibody (Santa Cruz). After stripping, the membranes were incubated with 20% methanol, 80% Tris-buffered saline for 5 min, then incubated with 0.5 mM 2,4-dinitrophenylhydrazine (DNP, Sigma) to detect carbonyl groups associated with aldehydes and ketones for 30 min at room temperature. The membranes were washed and then incubated overnight in anti-DNP antibody (Merck Millipore, Billerica, MA, USA), as previously described [33].

### Statistical analysis

The results were expressed as the mean ± standard deviation. Comparisons between groups were performed using an analysis of variance followed by Fisher’s protected least-significant difference test. The analyses were performed using StatView software (SAS Institute Inc., Cary, NC). P<0.05 was considered significant.

## Results

The characteristics of the db/m and db/db mice treated or not with EOC or EOC-CM infusions and non-diabetic or diabetic WKY rats are presented in Table 1.

**Table 1:** Physiologic parameters of the studied animals treated or not with EOC or ECO-CM intravenous injections.

Animals	Body weight	Blood glucose
	(g)	(mmol/l)
db/m (n = 5)	26.6 ± 1.8	5.76 ± 1.0
db/db + DPBS (n = 4)	45.5 ± 4.9 *	26.2 ± 9.3 †
db/db + EBM2 (n = 8)	48.0 ± 2.8 *	22.4 ± 8.8 †
db/db + EOC (n = 5)	48.0 ± 3.0 *	21.6 ± 9.5 †
db/db + EOC-CM (n = 10)	47.1 ± 4.2 *	21.0 ± 5.7 †
WKY-CT (n = 5)	413,2 ± 66.9	8.7 ± 0.8
WKY-DM (n = 4)	258,8 ± 39.6 **	31 ± 1.8 ††
WKY-DM+EBM2 (n = 4)	254.4 ± 35.6 **	30 ± 1.5 ††
WKY-DM+EOC-CM (n = 4)	230,1 ± 19.9 **	31.4 ± 1.3 ††

*P <0.0001 and

† *P* <0.0006 vs db/m

** *P*<0.0002 and

††*P*<0.0001 vs WKY-CT.

**Fig 1:**
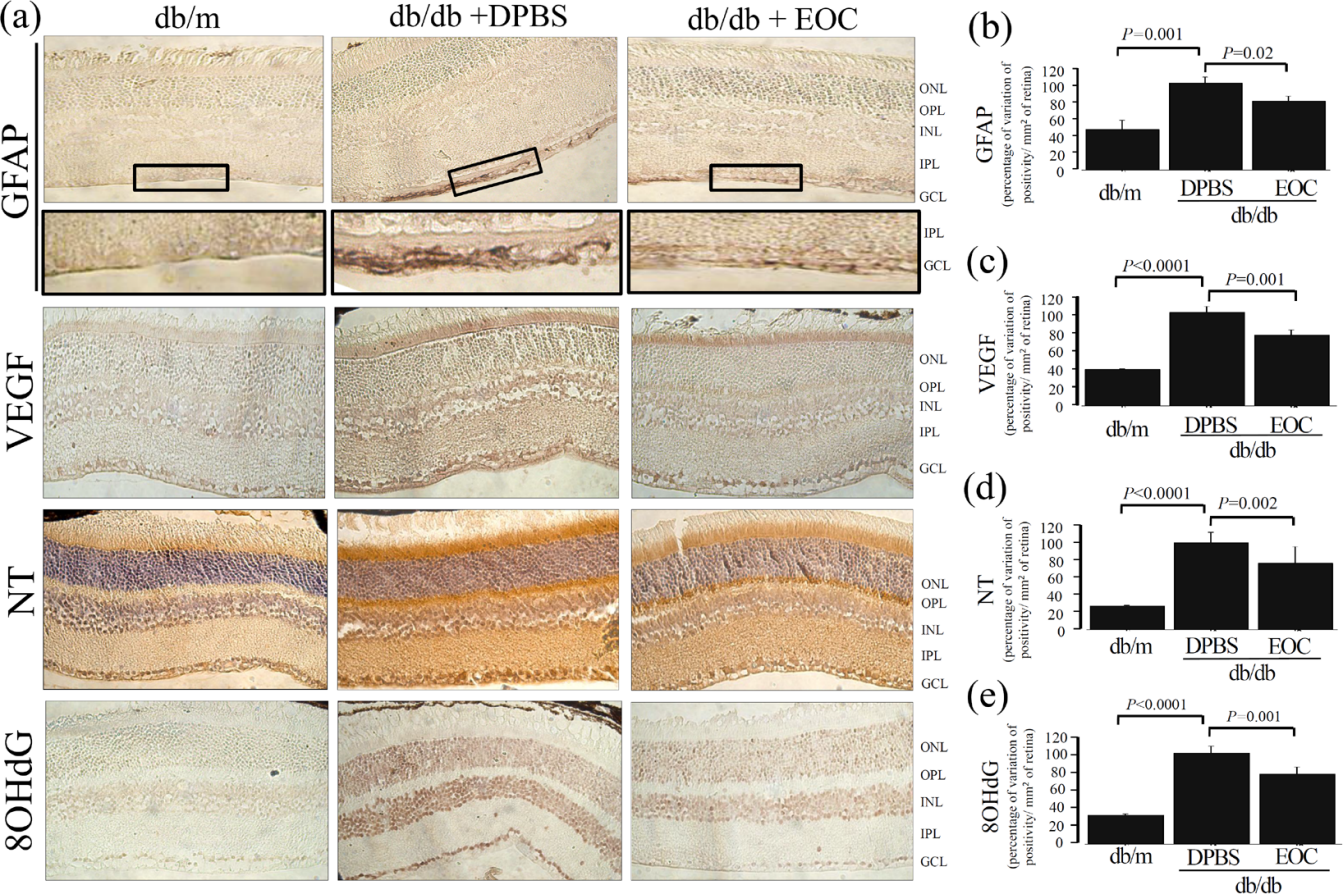
Intravenous injection of EOCs prevented diabetic retinopathy and oxidative retinal damage markers in db/db mice. (a) Representative photomicrograph of glial reactivity (GFAP), VEGF, nitrotyrosine (NT) and 8-OHdG in retinal tissue from mice treated with EOC. Magnification X400. (b-e) Semiquantitative analyses of immunolabeling.The semi quantitative analyses were performed using the Leica Application Suite (Leica Microsystems, Wetzlar, Germany) in nine non-consecutive retinal sections divided among three slides per animal, per group, under high-powered microscopic viewing (×400) (Zeiss, Jena, Germany).

**Fig 2:**
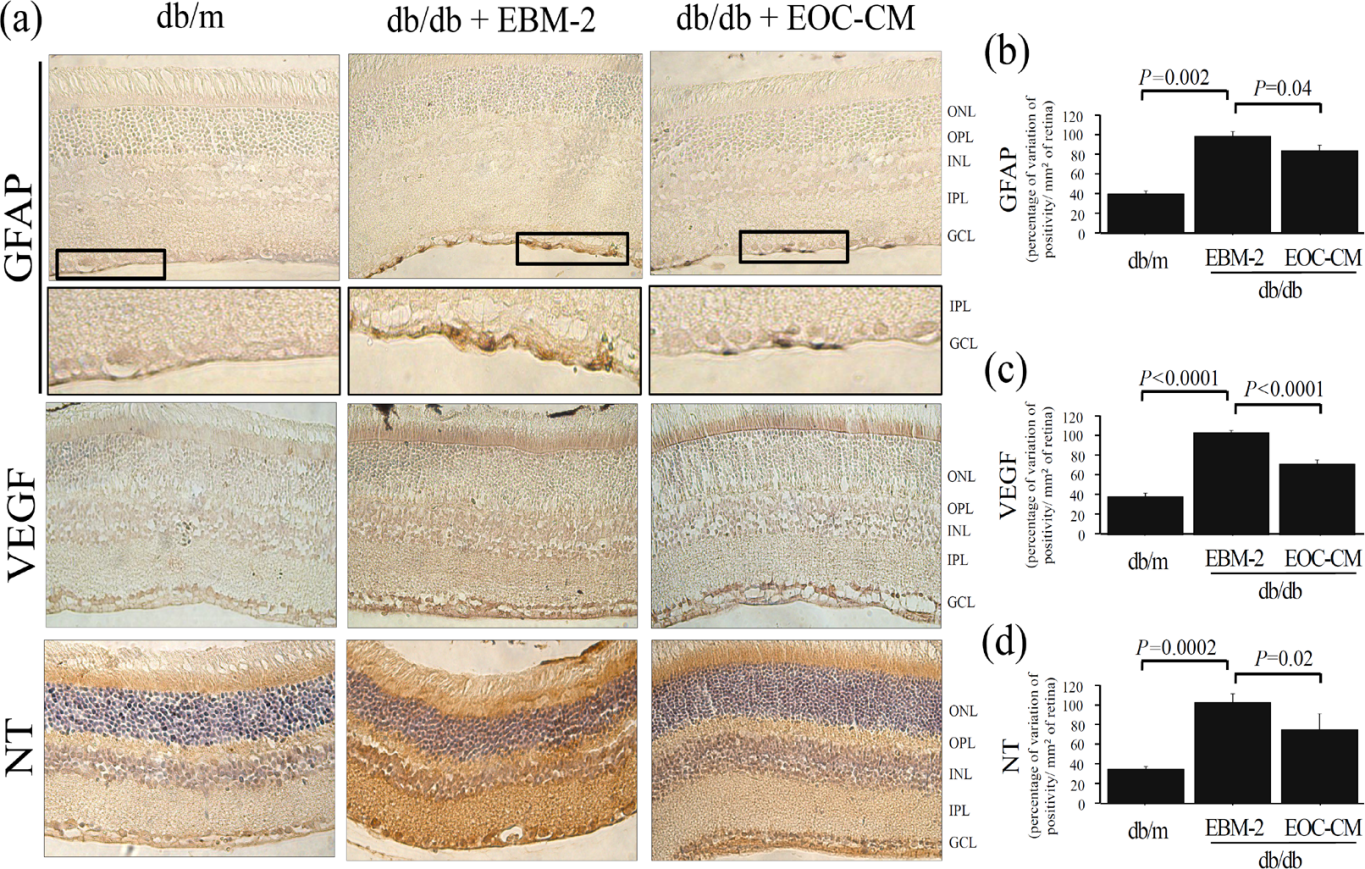
Intravenous injection of EOC-CM prevented retinopathy and oxidative damage markers in retina from diabetic mice. (a) Representative photomicrograph of astroglial reactivity (GFAP), VEGF and nitrotyrosine (NT) in retinal tissue from mice treated with EOC-conditioned medium (EOC-CM). Magnification X400. (b-d) Semiquantitative analyses of immunolabeling. Semiquantitative analyses were performed. The semi quantitative analyses were performed using the Leica Application Suite (Leica Microsystems, Wetzlar, Germany) in nine non-consecutive retinal sections divided among three slides per animal, per group, under high-powered microscopic viewing (×400) (Zeiss, Jena, Germany).

To investigate the effects of EOC administration on the diabetic retina, GFAP and VEGF expressions were assessed as markers of retinal glial reaction and early DR, respectively (Fig 1). The db/db non-treated mice expressed higher GFAP immunolabeling in the retinal ganglion cell layer compared to the db/m mice (P = 0.001) (Fig 1A and 1B). Similarly, there was an increase in VEGF immunoreactivity compared to the db/m mice (P<0.0001) (Fig 1A and 1C). Intravenous injection of EOCs prevented these alterations in the diabetic retinas (P<0.02). Nitrotyrosine(NT) and 8-OHdG expressions were assessed as indices of nitrosative and oxidative stress tissue damage, respectively. There was a marked increase in NT and in 8-OHdG immunolabeling in retinal tissue from the db/db mice compared to the db/m control animals (P<0.0001) (Fig 1A and 1D and 1E).EOC intravenous infusion protected the diabetic retinas from the increment of NT and 8-OHdG expressions (P<0.02 compared with DPBS treated db/db mice).In order to demonstrate the endocrine mode of action of the cell therapy presented herein, diabetic mice were randomized to receive intravenous injections of db/m EOC-CM or unconditioned medium (Fig 2). The retinal protective effects of intravenous infusion of CM were similar to those observed in db/db mice treated with EOC infusion. The morphological alterations detected in diabetic retina as GFAP, VEGF and nitrotyrosinewere all prevented in db/db mice, which received the db/m EOC-CM therapy (P = 0.04, P<0.0001 and P = 0.02, respectively). This is a demonstration that the EOC therapy displays potent retinal protection under diabetic conditions and the beneficial effect is attributed to its paracrine capacity. For a better understanding of the underlying mechanism, an *in vitro* study was conducted.

**Fig 3:**
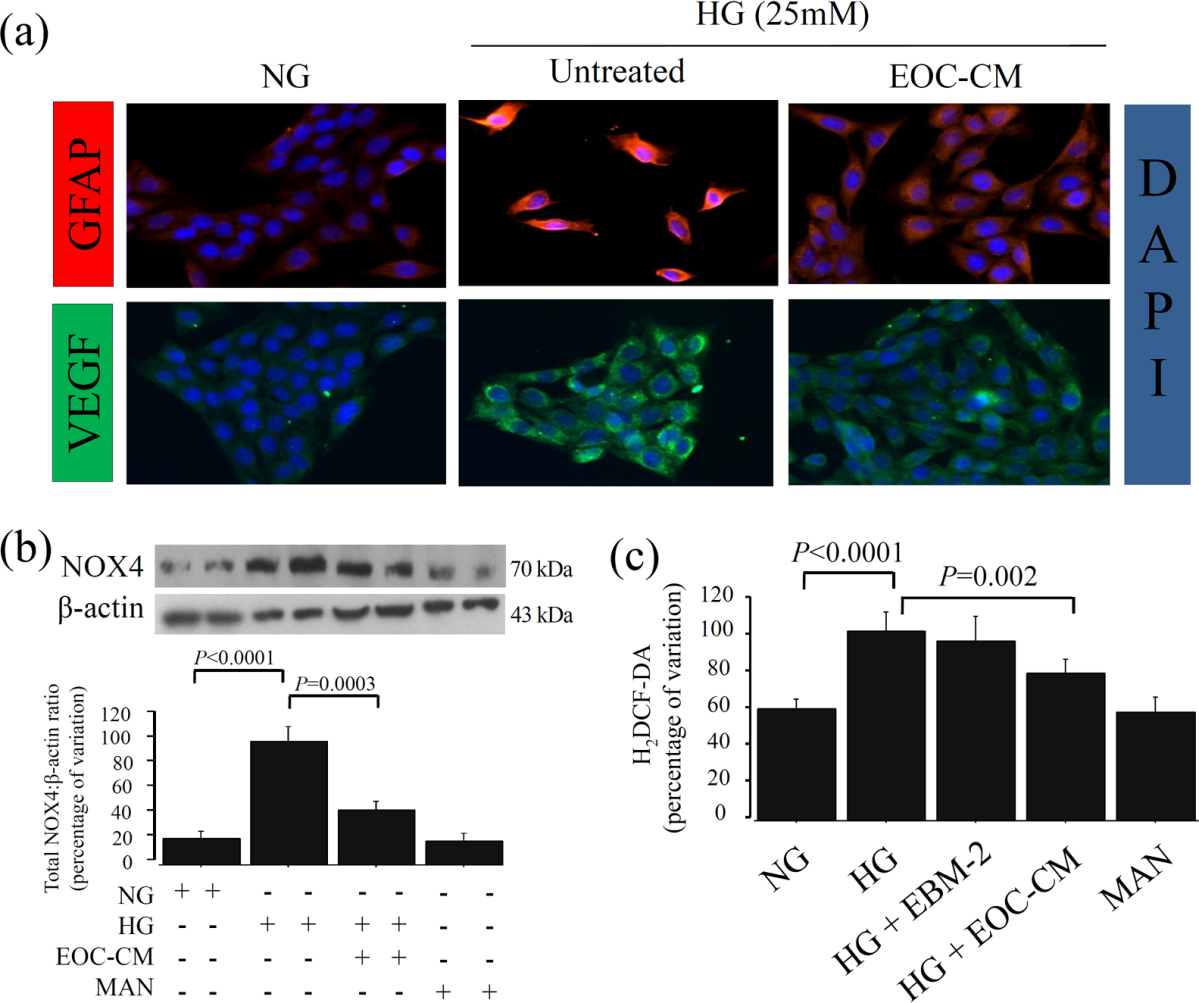
EOC-CM reduced GFAP and VEGF expressions and prevented glucose-induced ROS production by downregulating NOX4 expression in rMC-1 under diabetic milieu. (a) Representative photomicrograph of GFAP and VEGF immunofluorescence in rMC-1 cultured for 24 hours in high glucose (HG) in presence or not of EOC-CM; magnification: X630. (b) Representative Western blots for NOX4 in rMC-1.Equal loading and transfer were ascertained by reprobing the membranes for ß-actin. (c) The quantification of total intracellular reactive oxygen species (ROS) levels in rMC-1. Values were corrected by the number of cells at the end of each treatment.

To address the mechanisms by which EOCs or their CM prevented the diabetic milieu abnormalities observed in retinal tissue, rMCs were exposed to HG condition in presence or not of db/m EOC-CM. As observed in the *in vivo* study, EOC-CM was efficient in preventing GFAP and VEGF alterations (Fig 3A). Since these structural abnormalities were accompanied by oxidative stress damage in retinal tissue, we assessed NOX4 expression, a subunit of NADPH oxidase that is highly correlated with chronic diabetic complications.[34,35] rMCs under HG display a higher expression of NOX4 compared to NG conditions, which was significantly reduced by EOC-CM treatment (P = 0.0003) (Fig 3B). As a consequence, the production of total reactive oxygen species (ROS) was dramatically reduced in rMCs exposed to diabetic milieu in presence of EOC-CM (P = 0.002) (Fig 3C). These findings indicate that EOC administration exerts antioxidant effects in retinal cells exposed to HG through amelioration of the NADPH oxidase pathway.

**Fig 4:**
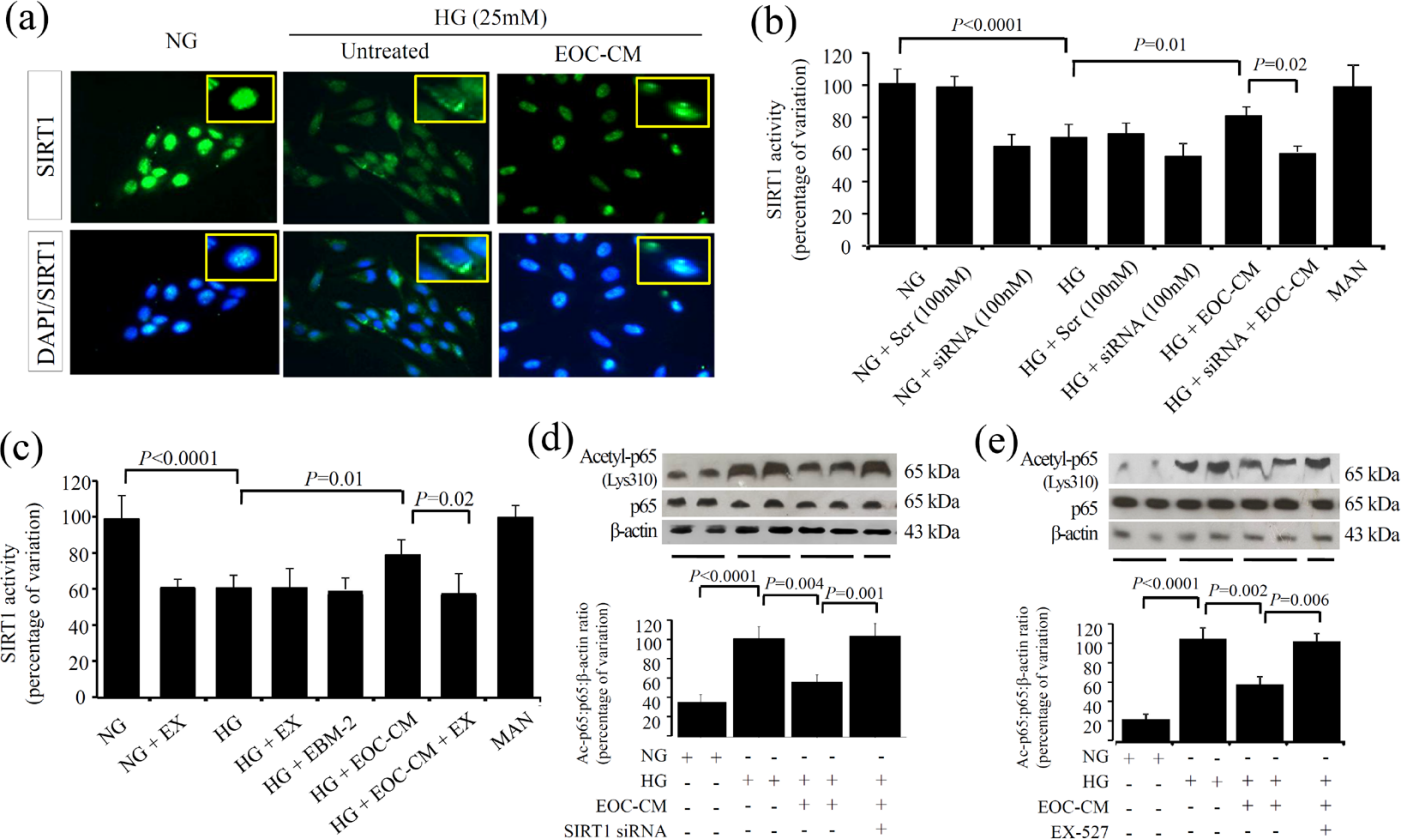
EOC-CM improved SIRT1 deacetylation pathway in rMC-1 exposed to high glucose conditions. (a) Immunofluorescence for SIRT1 in rMC-1 cultured for 24 hours. In normal glucose (NG), the SIRT1 signal was stronger and present in the nucleus; in high glucose (HG), the staining becomes faint and appears to be located predominantly in the cytosol; magnification X1000. (b-c) SIRT1 activity in rMC-1 cultured for 24 hours under different conditions was estimated by the fluorescent method. (d-e) Representative Western blots for acetyl-Lys310-RelA/p65 in rMCs-1 exposed to HG in presence or not of EOC-CM combined or not with SIRT1 siRNA (d) or EX-527 (e). Equal loading and transfer were ascertained by reprobing the membranes for ß-actin.

**Fig 5:**
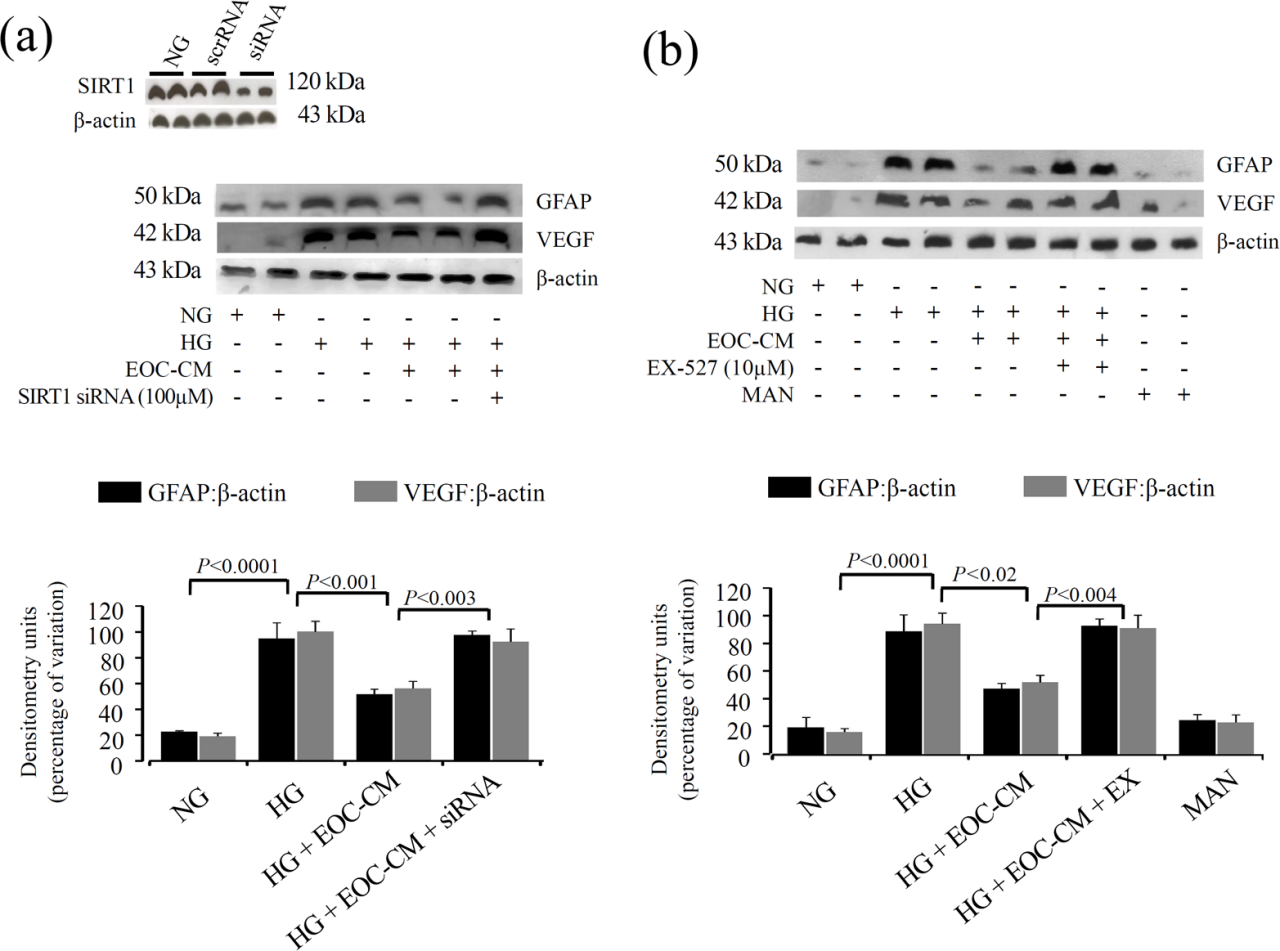
GFAP and VEGF proteins were downregulated by EOC-CM in rMC-1 under diabetic conditions through SIRT1 signaling. (a-b) Representative Western blots in total cell lysate against GFAP and VEGF. Equal loading and transfer were ascertained by reprobing the membranes for ß-actin.rMCs-1 exposed to HG and treated with EOC-CMs in presence or not of SIRT1 siRNA (a) or EX-527, a SIRT1 inhibitor, (b). The efficiency of the SIRT1 siRNA(100nM) was ~59%.

**Fig 6:**
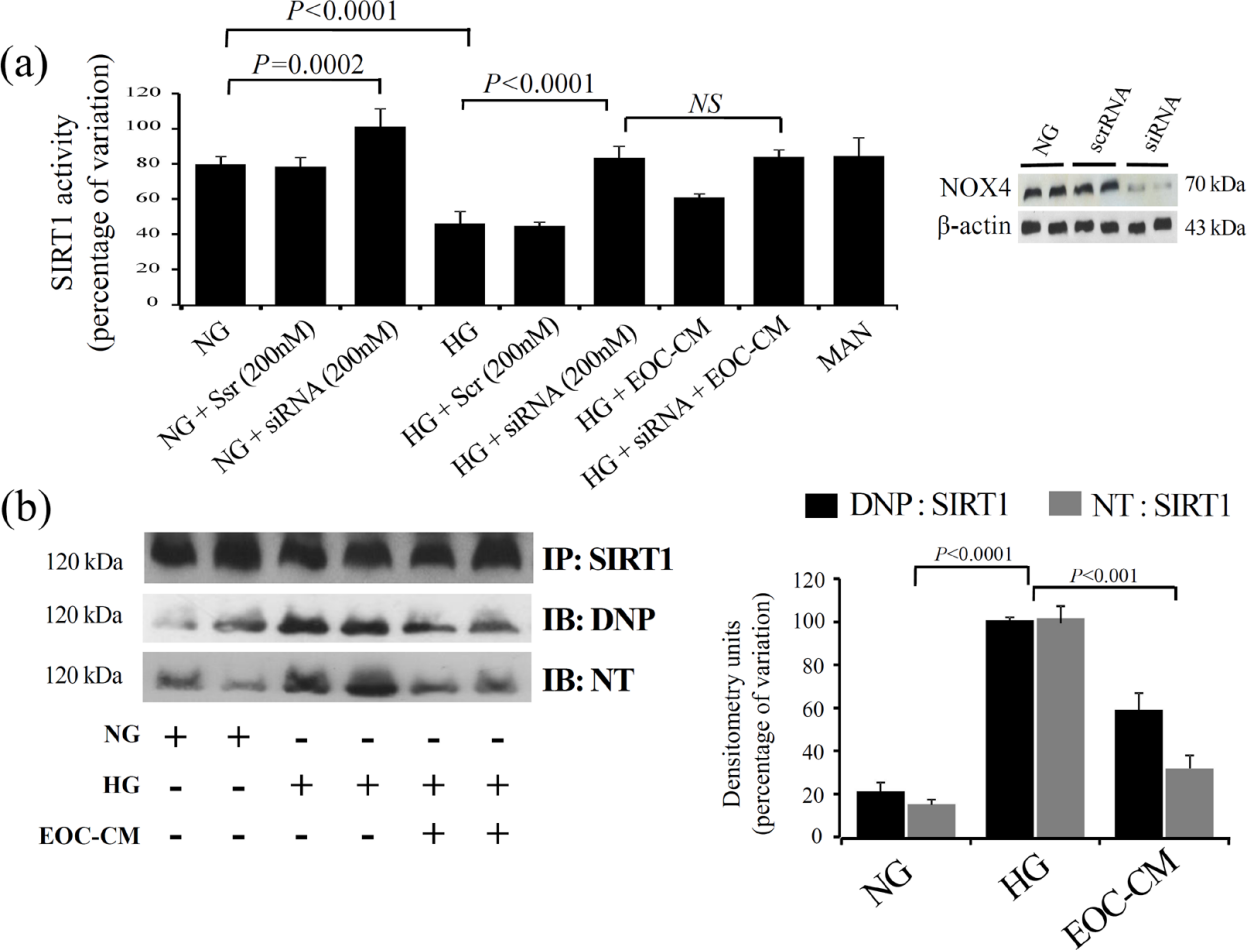
EOC-CM prevents SIRT1 post-translational modification and improved SIRT1 activity throughNOX4–NADPH oxidasedownregulation in rMC-1. (a) SIRT1 activity in rMC-1 cultured for 24 hours by a fluorescent method. Cells in NG or HG conditions were exposed to NOX4siRNA in presence or not of db/m EOC-CM or db/db EOC-CM. Representative Western blots for NOX4 in rMC-1 total lysate cultured for 24 hours in HG. The efficiency of the NOX4small interfering RNA (100nM) was ~63%.NS = no significant difference. (b) SIRT1 immunoprecipitation in rMCs-1 lysates exposed to HG in presence of EOC-CM. Carbonylation was detected by first derivitizing the samples with DNPH and immunoblotting with DNP antibody. Nitration was detected by immunoblottingwith nitrotyrosine (NT) antibody.

As oxidative stress is the upstream event in sirtuin regulation, we assessed the SIRT-1 expression and activity in rMCs under diabetic conditions. An immunofluorescence assay revealed that SIRT1, which is a nuclear factor in its active form, is highly expressed in normal conditions. HG exposure to rMCs induced a marked decrease in the nuclear expression accompanied by translocation to cytosol; treatments with EOC-CM re-established SIRT1 nuclear positivity (Fig 4A). SIRT1 activity evaluated through a fluorometric assay revealed that rMC-1 cells exposed to the HG conditions displayed lower SIRT1 activity (P < 0.0001). The presence of EOC-CM prevented this abnormality (P = 0.01) (Fig 4B). To demonstrate the role of SIRT-1 activation on EOC-CM treatment effects, the rMCs were treated with siRNA for SIRT-1 (Fig 4B and 4D) orwith specific inhibitor EX-527 (Fig 4C and 4E). Cells exposed to HG conditions displayed an increase in total acetylated lysine protein with a 2.8-fold increment of Lys310-RelA/p65 acetylation (P<0.0001). The treatment with EOC-CM caused a decrease in this level (P = 0.004 and P = 0.002, respectively) indicating a significant improvement in SIRT1 activity. The presence of the siRNA or SIRT1 inhibitor thoroughly abolished this effect. As expected, the presence of either siRNA or EX-527 abolished the effects observed in rMC exposed to HG leading to increment of p65-NFkB subunit acetylation (Fig 4D and 4E, respectively). As observed in the *in vivo* study, EOC-CM was efficient in preventing GFAP and VEGF alterations present in rMCs under diabetic milieu (Fig 5A and 5B). As demonstrated that NOX4-NADPH subunit is upregulated in rMCs exposed to HG conditions, we aimed to investigate the cross-talk between SIRT-1 and NOX4-NADPH-oxidase systems. In rMCs under HG conditions, the silencing of NOX4 gene incited an increment of about 25% SIRT1 activity (P < 0.0001) (Fig 6A); the combination with CM did not add further effect. This finding indicates that SIRT1 is pivotal in EOC-CM mechanism of action, and that the protective are at least partly mediated by re-establishment of NOX4-NADPH-oxidase subunit.Finally, in order to further understand the mechanism by which EOC-CMs modulate SIRT1 activity, we assessed the possible posttranslational modification of SIRT1 protein in the HG condition. rMCs exposed to HG displayed a significant increase in SIRT1 carbonylation and nitrosylation modifications (P<0.0001) (Fig 6B). The treatment with db/m EOC-CMs decreased these modifications (P<0.0003), thus indicating that EOC-CM prevents carbonylation and nitration modifications on SIRT1 moleculesin diabetic milieu conditions.

**Fig 7:**
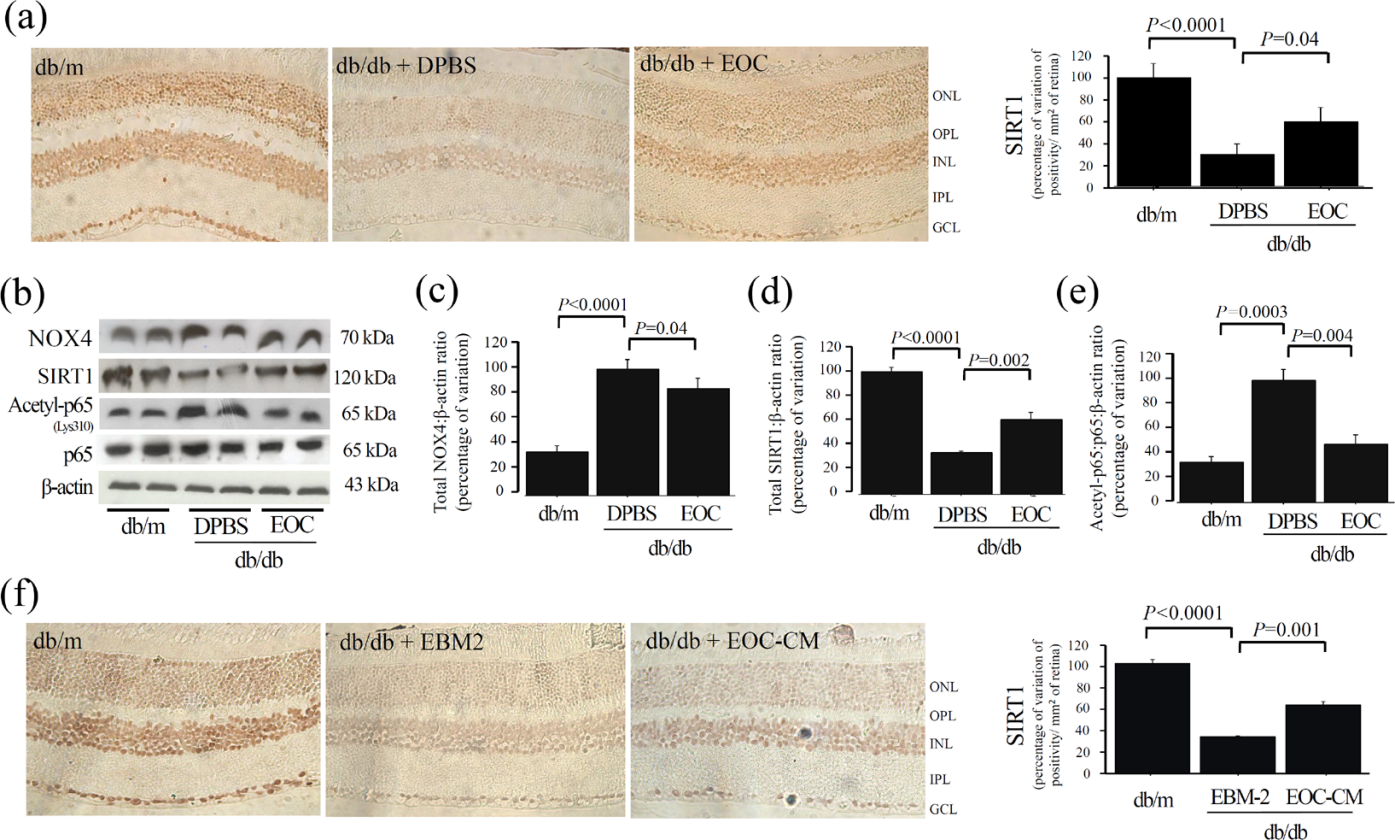
EOC or EOC-CM intravenous injections treatments improve retinal Sirt1 pathway in db/db mice. (a) A representative photomicrograph of immunolocalization of Sirt-1 in retinal tissue from mice treated with EOC. (b) Western blot for NOX4, Sirt1 and acetyl-Lys310-p65 subunit-NFkBexpression in mice retinal tissue lysate. Equal loading and transfer were ascertained by reprobing the membranes for ß-actin. (b-e) The arbitrary unit of densitometry was transformed to folders of increment in relation to the media of db/m in each experiment to compare independent experiments. (f) A representative photomicrograph of immunolocalization of Sirt1 in retinal tissue from mice treated with EOC-conditioned medium (EOC-CM).

As a proof of concept, the expression of SIRT1 was verified in retinal tissues from db/db mice. Diabetic mice treated with either EOCs (Fig 7A) or their CM (Fig 7B) via intravenous infusion displayed a marked improvement inSIRT1 retinal expression. This phenotype is accompanied by improvement of NOX4 and SIRT1 deacetylation of Lys 310-p65 NFkB subunit (Fig 7A–7F).

**Fig 8:**
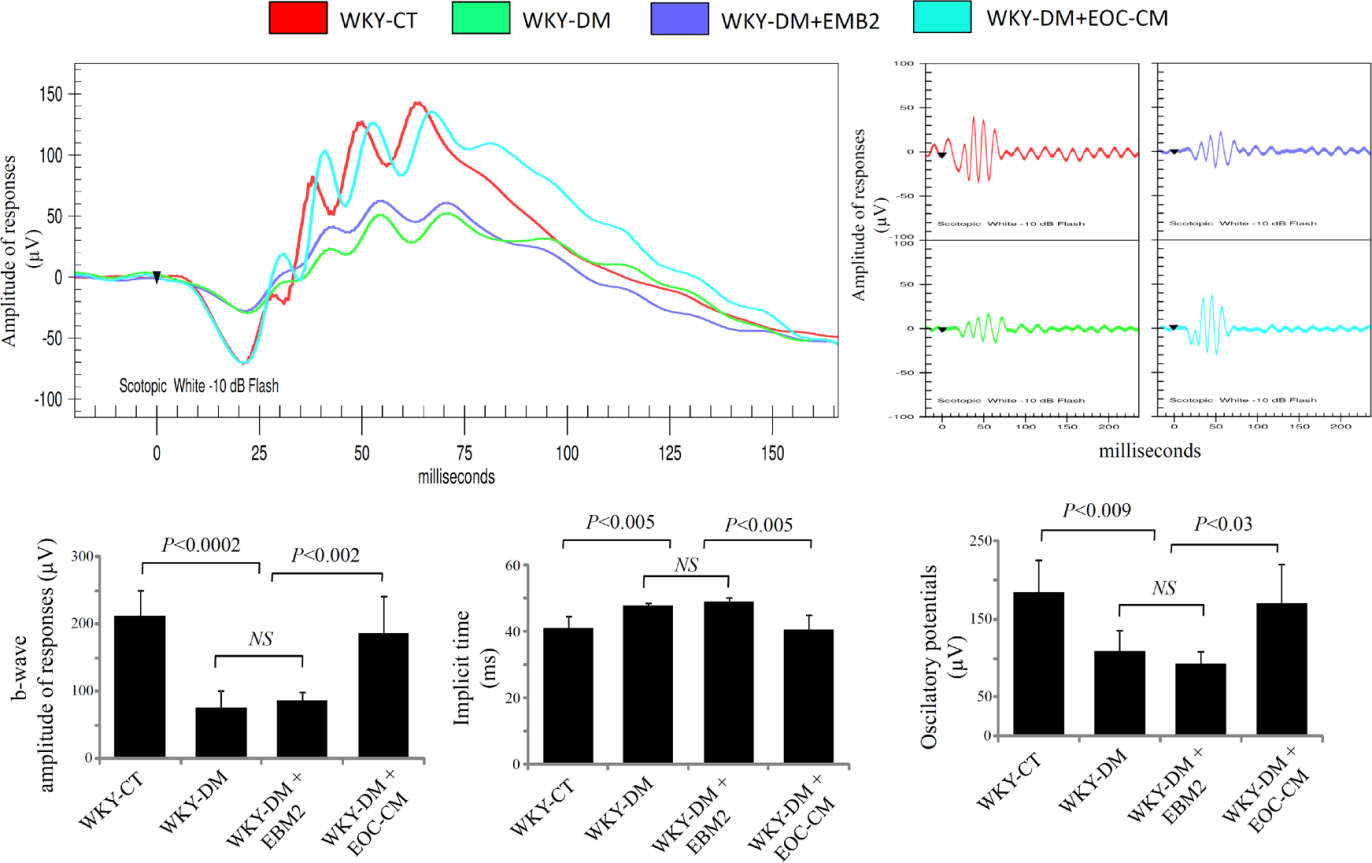
EOC-CM intravenous injection treatment restores retinal function in diabetic WKY rats. Representative waveforms of *b*-waves and oscillatory potentials in the studied rats. The *b*-wave is the positive deflection generated in part by the Müller and mainly by the bipolar cell potentials. The figure shows representative full-flash ERG waveforms and oscillatory potentials at 0.25 cd.s/m^2^ for a non-diabetic (WKY-CT, red), diabetic untreated (WKY-DM, green), diabetic treated with EBM2 medium (WKY-DM+EMB2, purple) and diabetic treated with EOC-conditioned medium (WKY-DM+EOC-CM, blue). The bars represent mean±S.D. of *b*-wave ampliutude (μv), implicit time (ms) and oscillatory potentials (μv) from all animal groups.

To determine whether EOC-CM is capable of preventing visual disturbances in diabetic rats, retinal function was estimated through full-flash ERG. Diabetic WKY rats showed a significant decrease in the amplitude of the *b*-waves and oscillatory potentials accompanied by increase in implicit time responses. This functional alteration on retinal responses reflects impairment of inner retinal layers. The intravenous injections of EOC-CM improved those parameters to normal levels (P<0.03), thus restoring the retinal function (Fig 8). This finding reveals that the endocrine capacity of EOCs may be a possible new therapeutic tool for DR treatment.

## Discussion

In this study, cell therapy using EOCs or using the CM either from db/m or from isogenic rat WKY cultured EOCs were shown to be potential therapies preventing of the early changes in DR. Both treatments prevented oxidative damage, avoided the glial reaction and VEGF expression levels increment. In diabetic WKY rats, the retinal function evaluated through eletroretinography was restored in cell-free therapy through intravenous injection of CM. As a lack of cell retention in injured tissue previously demonstrated [14] the high endocrine capacity of these cells allows them to act at distant sites. For retinal treatment, this is an advantage, since no direct manipulation of the eye is needed. The mechanistic pathways involved in these effects were studied in cultured retinal Müller cells. The rMCs exposed to HG treated with EOC-CM showed improvement in SIRT1 activity through modulation of the NOX4–NADPH oxidase subunit, thus reducing ROS formation. This process led to the deacetylation of Lys310-p65, which downregulated the expression of VEGF and GFAP proteins. The complete proteome analyses of the conditioned medium from EOCs shown previously by Gilbert and collaborators [15] described approximately 300 proteins, including GST1, Prdx, Trx, and NADP^+^, which contribute to the antioxidant effects observed in EOC-treated mice. The observation that NOX expression/activity can be modulated by antioxidant enzyme defences is supported by previous work in renal cells, where the NOX subunit is simultaneously the target and agonist of TGF-β [36]. Indeed, most agents that improve renal pathology prevent the increase of NOX components [37]. The high antioxidant capacity of these cells, equilibrating the redox status, prevented the posttranslational modifications on the SIRT1 molecule from restoring its activity under diabetic conditions.

Since the landmark paper by Asahara [38], EPCs have been the center of regenerative medicine, mainly involving cardiovascular diseases and neovascularisation [39,40]. Herein, the treatment with systemic EOCs or their CM invoked protection of retinal tissue from diabetes insult due to its high antioxidant property, thus leading to reestablishment of SIRT1 activity. In conclusion, compelling evidence is provided that equally beneficial effects were observed in treatments using EOCs or their CM, promoting retinal protection from glucose-related oxidative damage. Further studies using conditioned media from EOCs are needed in order to provide evidences of possible efficiency for autologous EOC therapy in patients with diabetic retinopathy.

## Abbreviations

AGEs: advanced glycation end products
CD34^+^: cluster of differentiation 34
DR: diabetic retinopathy
DMEM: Dulbecco’s modified Eagle’s medium
EOCs: early outgrowth cells
EGM-2: endothelial growth medium-2
EGM-2: endothelial growth medium-2
EPCs: endothelial progenitor cells
EOC-CM: EOC-conditioned medium
GST1: glutathione S transferase
HSP: heat shock protein
HG: high glucose
MAN: mannitol
NOX4: NADPH oxidase 4
NAD^+^: nicotinamide adenine dinucleotide
NT: nitrotyrosine
(NF)κB-p65: nuclear factor
NT: Nytrotyrosine
Prdx: peroxiredoxin
Prdx: peroxiredoxin
GFAP: protein glial fibrillary acidic protein
rMCs: Rat retinal Müller cells
ROS: reactive oxygen species
RPE: retinal pigment epithelium
Scr: scrambled siRNA
SIRT1: silent information regulator 1
SIRTs: Sirtuins
siRNA: small interfering RNA
siRNA: small interfering RNA
SOD: superoxide dismutase
SOD2: superoxide dismutase-2
MTT: thiazolyl blue tetrazolium bromide
Trx: thioredoxin
Txn: Trx-interacting protein
TGF-β: transforming growth factor beta
VEGFR2^+^: vascular endothelial growth factor receptor 2
WKY: Wystar Kyoto rat
